# The indirect impact of the technostress subfactors on the satisfaction and desire to work from home

**DOI:** 10.3389/fpsyg.2024.1417916

**Published:** 2024-06-12

**Authors:** Adél Csenge Simon, Barnabás Buzás, Orsolya Rosta-Filep, Klára Faragó, Orsolya Csilla Pachner, Orhidea Edith Kiss

**Affiliations:** ^1^Doctoral School of Psychology, Eötvös Loránd University, Budapest, Hungary; ^2^Institute of Psychology, ELTE Eötvös Loránd University, Budapest, Hungary; ^3^Institute of Education and Psychology at Szombathely, ELTE Eötvös Loránd University, Budapest, Hungary

**Keywords:** technostress, working from home, organizational digitalization, latent deprivation model, WFH intention

## Abstract

**Introduction:**

Organizational digitalization is a phenomenon that is becoming more widespread and holistic; that is responsible for more employees being affected by digital work and working from home. While introducing remote work offers numerous economic benefits for organizations, this transition must be aligned with employees' needs rather than in an authoritarian manner. Our research aimed to investigate how sub-factors of technostress, directly and indirectly, influence the satisfaction and desire to work from home.

**Methods:**

We conducted a cross-sectional survey with a sample of 361 office workers with at least two years of experience who have spent some time working from home. We checked our hypotheses with a path model.

**Results:**

Our research found that techno-insecurity and techno-complexity have a minimal direct influence on the desire to work from home. However, the desire to work from home significantly decreases through various mediation pathways via the status sub-factor (which can be seen as one of the latent benefits of remote work) and through satisfaction with working from home. Our model explains 33.7% of the variance in the desire to work from home.

**Discussion:**

This suggests that leaders have a task of great significance: to decrease the technostress employees are exposed to and to draw the attention of researchers to the fact that technostress has more complex indirect effects than previously assumed.

## 1 Introduction

### 1.1 Background

Digitalization is a fundamental transformation led by technology that affects all aspects of life. One of the areas most affected by the digitalization is the world of work (Dragano and Lunau, 2020). Organizational digitalization is a decades-long process responsible for the constant changes in work organization forms, tasks and work conditions (Cijan et al., 2019). One of the main driving forces of digital transformation is the assumption that introducing new technologies has a positive impact on innovation, efficiency and provides an economic competitive advantage (Solberg et al., 2020; Ballestar et al., 2021). Due to digitalization, work has transferred to new interfaces and devices; with the spread of the internet or smartphones, computers and laptops have started to gain ground in work life. Therefore working from home could also appear and constantly become a more popular form of work organization since the 1970s (Hackney et al., 2022).

There are several types of digital out-of-office working, such as home office and homeworking. The term home office refers to an employee working from home occasionally for a predetermined reason, while homeworking is a contractual approach whereby the employee works from home permanently, by mutual agreement between the employer and the employee (Beno, 2018). Unlike the previously mentioned work organizational forms, telecommuting allows employees to work from any location other than the office, facilitated by various platforms that support a flexible work schedule (ten Brummelhuis et al., 2012). Our current research does not distinguish between the various forms of remote working. Therefore, any work done outside of an office in the employee's home will be referred to as working from home (WFH) and examined as such. It is important to note that working from home and digital work are separate but closely associated terms because most employees who work from home or remotely rely on digital devices. Our research examines employees working digitally and from home.

Even though we are discussing a form of work organization that has existed for the past 50 years, every form of WFH has become widespread worldwide due to COVID-19 and the regulations it invoked. The pandemic affected 213 countries and territories; in its wake, millions lost their jobs or had to start working from home (Farooq and Sultana, 2021). Meanwhile, after the abolishment of the regulations, WFH's popularity has not diminished. This is shown in the Eurostat (2023) survey, which shows that in 2019, only 5.5% of employed Europeans worked from home; in 2022, this number rose to just 10.2%. WFH is slowly replacing working from an office and spreading as the new norm (Davis et al., 2020). It is important to note that the changes affect the various groups of the labor market differently.

WFH is now primarily available to higher-status, well-educated employees who no longer need an office setting (Garrote Sanchez et al., 2021; Piroşcă et al., 2021). At the same time, these employees and organizations do not only have the opportunity to WFH but also have a demand for it, as recent studies have shown (Baert et al., 2020; Beck et al., 2020; Olde Kalter et al., 2021; Stefaniec et al., 2022). As a result, digital work and WFH are gaining popularity as a new norm and are presented as a method of work organization in the labor market; more studies are focusing on its impact. WFH offers many advantages for employees, such as the disappearance of commuting and the stress it induces, more flexible working hours, the growth of independence and sometimes comfort, and the fact that it helps handle various tasks at work and in private life (Elst et al., 2017; Narbarte et al., 2020; Farooq and Sultana, 2021). In addition, organizations benefit from remote work as it allows them to optimize office space based on the new work structure. This leads to reduced office accommodation costs for employees, thereby lowering rent and overhead expenses (Elst et al., 2017; Grozdics et al., 2023).

Numerous studies focus on WFH's effects on work performance. Although most studies show that work performance decreases, it is clear that there are individual and task-level differences, so in some cases, work performance may improve (Aczel et al., 2021; Russo et al., 2021). The studies highlight that the efficiency of working from home is influenced by external ambient conditions (Garrote Sanchez et al., 2021). If the employee possesses a calm, private area where they can retreat to work, then WFH may prove more effective than working from the office (Barrero et al., 2021). Similarly to work performance, the followers' voice may decrease due to WFH; however, the adverse effects of this can be compensated by the leaders' openness (Buzás and Faragó, 2023). These results suggest that digital working may have much more complex organizational implications than previously thought.

At the same time, the emergence of technostress can be seen as a consequence of digital working that affects all employees. Technostress is the term used to describe all kinds of work stress related to digital work (Dragano and Lunau, 2020). Craig Brod first noted the phenomenon that computer devices may cause stress in users. He defined technostress based on his clinical experience, a “modern disease of adaption caused by an inability to cope with the new computer technologies in a healthy manner” (Brod, 1984). In later studies, Brod's definition was refined, and its symptoms were determined, such as panic, anxiety, technophobia, mental fatigue, perfectionism, muscle cramps, headaches, joint pain and insomnia (Champion, 1988; Çoklar and Sahin, 2011; La Torre et al., 2020).

Technostress is a complex psychological construct which can be separated into multiple subfactors. There is no universally accepted classification currently, but recent academic articles regard the Technostress Creators as the standard as defined by Tarafdar (Berg-Beckhoff et al., 2017; Dragano and Lunau, 2020; La Torre et al., 2020). In this model, the subfactors: techno-overload, techno-complexity, techno-insecurity, techno-uncertainty and techno-invasion are included (Tarafdar et al., 2007). By techno-overload, we mean that working with digital technologies is demanding due to the high pace of work, frequent interruptions, multitasking, longer working hours and the expectations regarding the response time for digital communication. In the case of techno-invasion, the lines separating work and private life become blurry due to the flexibility that digital devices allow us. Techno-complexity is a phenomenon we experience when new digital technologies are perceived by employees as highly complex and, therefore, challenging to master. Techno-insecurity is a fear of losing one's job or status because the employees assume that new digital technologies or more skilled workers can take over their duties. Techno-uncertainty refers to an uncertainty caused by chronic digital transformation processes or the constant change of a single technology (Ragu-Nathan et al., 2008; Dragano and Lunau, 2020; Torres, 2021).

Numerous studies explored the harmful effects of technostress in the past decades. One of the first studies looked at the onset of technostress in librarians. It revealed that at the birth of electronic libraries, librarians reacted to introducing new digital technologies by displaying behaviors such as resistance to tools and training. The number of absences and late arrivals increased (Bichteler, 1987). When researchers attempted to identify the factors behind this phenomenon, they found that the pace of technological innovation was too quick, which increased the workload, while the new tools were not yet reliable (Ennis, 2005).

In the 40 years since technostress was first defined, numerous studies have focused on the phenomenon, showing that it is associated with demographic variables (age, gender, educational attainment) and organizational psychological variables such as a decline in organizational commitment, increased role ambiguity, work-life imbalance, increased job insecurity, decreased job satisfaction and increased job exhaustion, and changes in individual performance (Salazar-Concha et al., 2021). In the meantime, it is essential to point out that a few recent studies have reported positive changes in the wake of technostress; in some cases, it can aid innovation and improve performance (Tarafdar et al., 2019).

Nevertheless, WFH may pose a problem for employees, not just with the onset of technostress, but it can make it more difficult for them to experience the latent benefits of work. For decades, it has been a well-known fact in organizational psychology that employees do not only work for their salary but also for the many positive benefits of work. In 1982, Marie Jahoda published her latent deprivation model, claiming that losing the five latent benefits of work (time structure, activity, social contact, collective purpose, and status) will worsen the employee's wellbeing (Jahoda, 1982). Initially, the model was used to examine the deterioration of mental health in unemployed people (Jahoda, 1982; Paul et al., 2007; Paul and Batinic, 2010; Selenko et al., 2011; Muller and Waters, 2012; Aitken et al., 2021), but over the last decade, studies have been conducted on many other work groups regarding the model. In a recent study, Paul demonstrated that not only the unemployed suffer from the loss of the latent benefits but also the retired and homemakers (Paul et al., 2023). In our previous research, we managed to document that for home-based employees, time spent working from home indirectly impairs affective commitment to the workplace organization through latent factors (social contact and collective purpose) (Simon et al., 2023). All of this suggests that while the manifest benefit, the salary, remains even if the employee works from home, it becomes more challenging to experience the latent benefits, which should be addressed.

### 1.2 Aims of the current research

In our current research, we wanted to examine how the various subfactors of technostress affect satisfaction with WFH and the employee's intention to work from home through Jahoda's latent deprivation model. This research is of paramount importance because there is an ever-increasing demand for WFH both from the employees and the organization as well, due to the experiences gained from digital work that became widespread in the wake of the COVID-19 pandemic and the worldwide economic difficulties (for example, the increased maintenance costs for offline offices). Nevertheless, it is essential that organizations do not decree WFH for its employees one-sidedly but to adjust it to their needs, and for this, it is vital to know the negative effects digitalization has. Considering the anticipated results, we aim to develop future intervention programmes to reduce the negative impacts.

### 1.3 Hypotheses and analysis design

We conceptualized the research question in the form of a serial multiple mediation model, where techno-insecurity (X1) and techno-complexity (X2) are mediated by the latent function status (M1) and the satisfaction with WFH (M2) on the desire to work from home (Y). Although positioning the variables in a path model indicates a causal description of their relationship, we stress that the data underlying the analysis are cross-sectional; thus, we could not test causality. Path modeling is normally attended by this caveat (see Hayes, 2013, chapters 1 and 4 for a detailed discussion in the context of social psychology research); the attribution of causality is a matter related to research design and logic, and not to statistical inference (Cohen, 2013). Therefore, the present analysis emanates hypotheses consistent with theory and tests whether the collected data are consistent with the underlying argument. IBM SPSS Statistics 25 and AMOS 24 were used for data analysis.

We assumed that the two subfactors of technostress, techno-insecurity (X_1_) and techno-complexity (X_2_), indirectly affect the desire to work from home (Y) through the status subfactor described in Jahoda's latent deprivation model (M_1_) and through the satisfaction with working from home (M_2_). When testing our hypothesis, we wanted to investigate the impact of techno-insecurity and techno-complexity in relation to the status subfactor. As Califf and Springer (2022) pointed it out, these two technostress subfactors can threaten the social status of workers by jeopardizing their already acquired job position due to the difficulty of learning complex digital operations. Furthermore, a previous study has already shown that the fear of losing one's job due to digitalization is the biggest perceived stressor among IT workers. Therefore, it is of paramount importance to be the basis for further research (Satpathy et al., 2021). We hypothesized that the increased technostress while working from home may increase the fear of status deterioration, harming WFH satisfaction and thus indirectly eroding desire.

**Figure 1:**
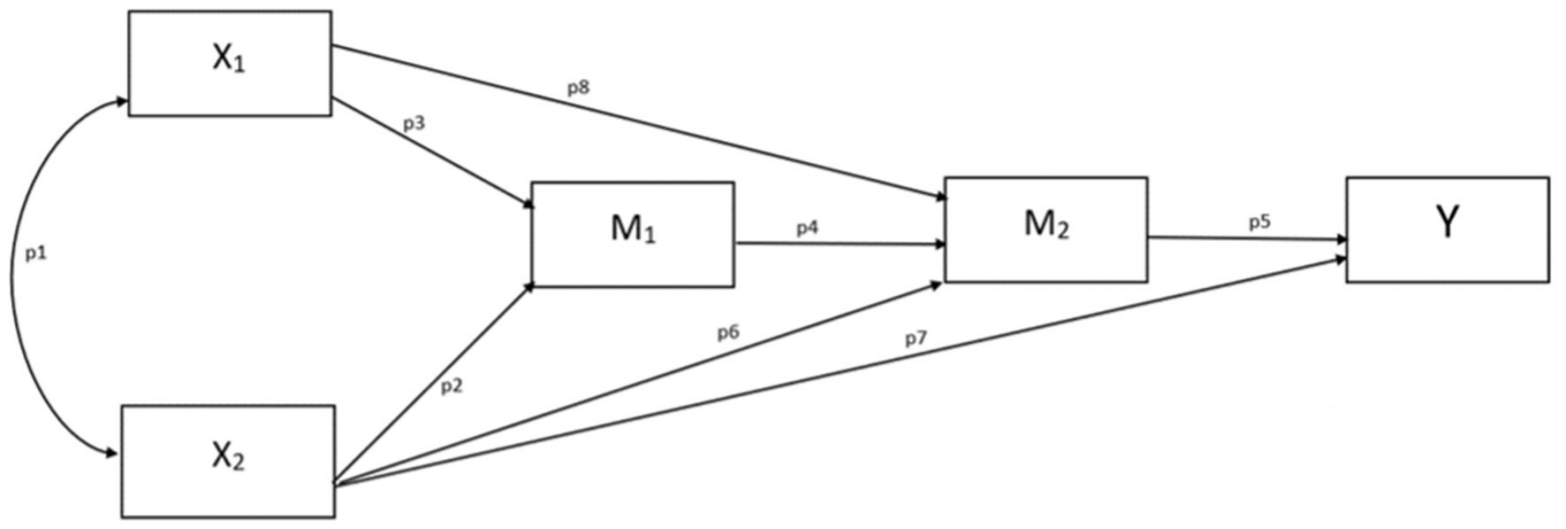
An outline of hypotheses, indirect effects of X_1_ and X_2_ on Y [techno-insecurity (X_1_), techno-complexity (X_2_) desire to work from home (Y) the status sub-factor of Jahoda's latent deprivation model (M_1_), satisfaction with working from home (M_2_)].

Our hypotheses are the following, which are also illustrated in Figure 1:

H1: We assume that techno-complexity (X_2_) reduces the desire to WFH (Y) through the status subfactor described in Jahoda's latent deprivation model (M_1_) and through satisfaction with WFH (M_2_). (p2^*^p4^*^p5)
H2: We assume that techno-insecurity (X_1_) reduces the desire to WFH (Y) through the status subfactor described in Jahoda's latent deprivation model (M_1_) and through satisfaction with WFH (M_2_). (p3^*^p4^*^p5)
H3: We assume that techno-complexity (X_2_) reduces the desire to WFH (Y) through satisfaction with WFH (M_2_). (p6^*^p5)
H4: We assume that techno-insecurity (X_2_) reduces the desire to WFH (Y) through satisfaction with WFH (M_2_). (p8^*^p5)

## 2 Methods

### 2.1 Data collection and participants

Participation was voluntary and anonymous; we used an online questionnaire for the data collection. Data was collected between March and April 2022. Ethical approval was given from Eötvös Loránd University, Budapest, Hungary (ELTE PPK; Reference number: 2022/94, Date: 25.02.2022). Criteria for inclusion included: (1) respondents had to have been employed for at least 2 years at the time of data collection; (2) they had to have spent at least part of their work time in WFH during the period between March 2020 (the first wave of COVID-19 in Hungary) and April 2022 and (3) they had to work in an office job. By specifying an office job, we wanted to ensure that our study sample included only workers who could work digitally.

Our survey included 361 respondents (231 women, 126 men, 4 who preferred not to say). 326 of the respondents were employees (90.3%), and 29 were entrepreneurs (8%). Looking at the study sample in terms of organizational hierarchy, 239 were subordinates (66.2%), 51 were middle managers (14.1%), 12 were senior managers (3.3%), and 17 were owners (4.7%). The average age of our study sample was 38.95 years (minimum = 20 years, maximum = 66 years, SD = 11.42 years). Of the employees who completed the questionnaire package, 126 worked in the public sector (34.9%) and 175 in the private sector (48.47%). Nonetheless, our sample cannot be perceived as representative of the whole Hungarian labor market. Notably, our research sample was restricted to employees who could work from home productively and were not forced to take a leave of absence.

Our study sample showed a heterogeneous distribution in terms of WFH. Since the outbreak of the COVID-19 pandemic and the beginning of the restrictions introduced in Hungary due to the pandemic (March 2020), the studied sample spent on average 47.7% (SD = 33.636%) of their working time WFH, while at the time of data collection, they spent on average 34.418% (SD = 34.141%) of their working time WFH. A similar result was found for the desire to work from home (question posed in the questionnaire: if you alone could decide what percentage of your working time to spend working from home, in what ratio would you work from home?), on average, it was 47.066% (SD = 28.9%). It is worth emphasizing the high standard deviation values for all three variables, which stress the significant variation in the sample and the heterogeneity in organizational and employee attitudes.

### 2.2 Scales

We used the following methods to measure the variables in the hypotheses.

#### 2.2.1 Work from home (WFH)

We measured the desire to work from home on scaling questions, where the employees were asked to indicate their responses as a percentage. The satisfaction with WFH was also measured with scaling questions on a 5-point Likert scale (“How satisfied are you with working from home?” 1 = I am not at all satisfied, 5 = I am very satisfied with it). There is no validated scale for measuring either satisfaction with WFH or the desire to work from home.

#### 2.2.2 Technostress creators inventory

We measured the various subfactors of technostress using the Technostress Creators Inventory questionnaire published by Ragu-Nathan in 2008 based on a model they had developed (Tarafdar et al., 2007; Ragu-Nathan et al., 2008). Since its publication, it has been the basis for many studies and has been validated in multiple languages (Dragano and Lunau, 2020; Torres, 2021; Kotek and Vranjes, 2022), although no Hungarian translation is available. The questionnaire consists of 23 items and is divided into 5 sub-factors. The items are rated by respondents on a 5-point Likert scale (1 = strongly disagree, 5 = strongly agree). The techno-overload (5 items) measures whether employees feel that digital work makes them work more and faster (“I am forced by this technology to work much faster”). Techno-invasion (4 items) is when employees feel that the constant availability due to digital technologies means that work is invading the rest of their lives (“I spend less time with my family due to this technology”). In the subfactor of techno-complexity (5 items), we studied how difficult it is for employees to master the technologies required for digital work (“I need a long time to understand and use new technologies”). The techno-insecurity (5 items) subfactor measures the extent to which employees fear losing their jobs because they do not have a high enough level of digital technology skills (“I have to constantly update my skills to avoid being replaced”). Techno-uncertainty (4 items) describes the stress caused by the constant digital transformation (“There are constant changes in computer hardware in our organization”). Table 1 shows the original and respective Cronbach's α values of the subfactors, which indicates that all of the scales had a satisfactory internal consistency.

**Table 1:** Reliability results for the technostress sub-factors.

**Subfactors**	**Original Cronbach α (Ragu-Nathan et al., 2008)**	**Respective Cronbach α**
Techno-overload	0.82	0.822
Techno-invasion	0.80	0.836
Techno-complexity	0.77	0.831
Techno-insecurity	0.78	0.805
Techno-uncertainty	0.83	0.840

#### 2.2.3 Latent and manifest benefits (LAMB) scale

The latent functions were measured using the Latent and Manifest Benefits (LAMB) Scale (Muller et al., 2005). The questionnaire was based on Jahoda's latent benefits theory (time structure, activity, social contact, collective purpose, and status) and Fryer's manifest benefits (salary) model. In the original version of the questionnaire, 36 items are featured; however, in our research, we relied on the shortened version published by Kovacs, where all 6 subfactors were measured with 3 items (Kovacs et al., 2019). The factors were measured using a seven-point Likert scale. Our current research only included the status dimension (Cronbach's α = 0.874) of the measured latent benefits in our mediation model. The status sub-factor measures the position in society that the job provides to the individual (e.g. “I am often valued by the people around me”) (Jahoda, 1982; Paul et al., 2007).

## 3 Results

Prior to hypothesis testing, confirmatory factor analysis (CFA) was used to test the reliability of the Technostress Creators [$\chi_{\left(220\right)}^{2}$ = 924.237 *p* < 0.01; CFI = 0.929; TLI = 0.919; RMSEA = 0.094] and the LAMB questionnaire [$\chi_{\left(120\right)}^{2}=$403.928 *p* < 0.01; CFI = 0.980; TLI = 0.975; RMSEA = 0.081]. The conducted tests/trials confirmed that the questionnaires measured accurately and that the studied variables were suitable for hypothesis testing. We examined the correlation between variables incorporated in the test model with the Spearman correlation; its results and the descriptive statistics of variables can be found in Table 2. We used the averaged results of the techno-complexity, techno-insecurity from the tested variables and the status sub-factor of Jahoda's latent deprivation model. Satisfaction with WFH was measured ordinally, so we cannot report the mean and standard deviation for this variable (Mode = 3, Median = 3); therefore, the distribution of responses can be seen in Table 3.

**Table 2:** The descriptive statistics and correlation analysis of the variables included in the test model (^**^means *p* < 0.001).

	**Descriptive statistics**		**Techno-insecurity (X_1_)**	**Techno- complexity (X_2_)**	**Status (M_1_)**	**Satisfaction with WFH (M_2_)**	**Desire to WFH (Y)**
	**M**	**SD**					
Techno-insecurity (X_1_)	1.943	0.64	-				
Techno-complexity (X_2_)	2.129	0.707	0.510^**^	-			
Status (M_1_)	5.48	0.975	−0.248^**^	−0.231^**^	-		
Satisfaction with WFH (M_2_)			−0.213^**^	−0.269^**^	0.200^**^	-	
Desire to WFH (Y)	47.06%	28.9%	−0.094	−0.242^**^	0.094	0.578^**^	-

**Table 3:** The distribution of responses to the question: “how satisfied are you with working from home?”

**Satisfaction with WFH**	**Frequency**	**Percent**
1. I am not at all satisfied	16	4.43%
2. I am rather dissatisfied with it	32	8.86%
3. I am both satisfied and not satisfied	106	29.363%
4. Rather satisfied	117	32.41%
5. Very satisfied	90	24.93%

According to preliminary assumptions, there is a positive, moderately strong relationship between the two sub-factors of technostress.

**Figure 2:**
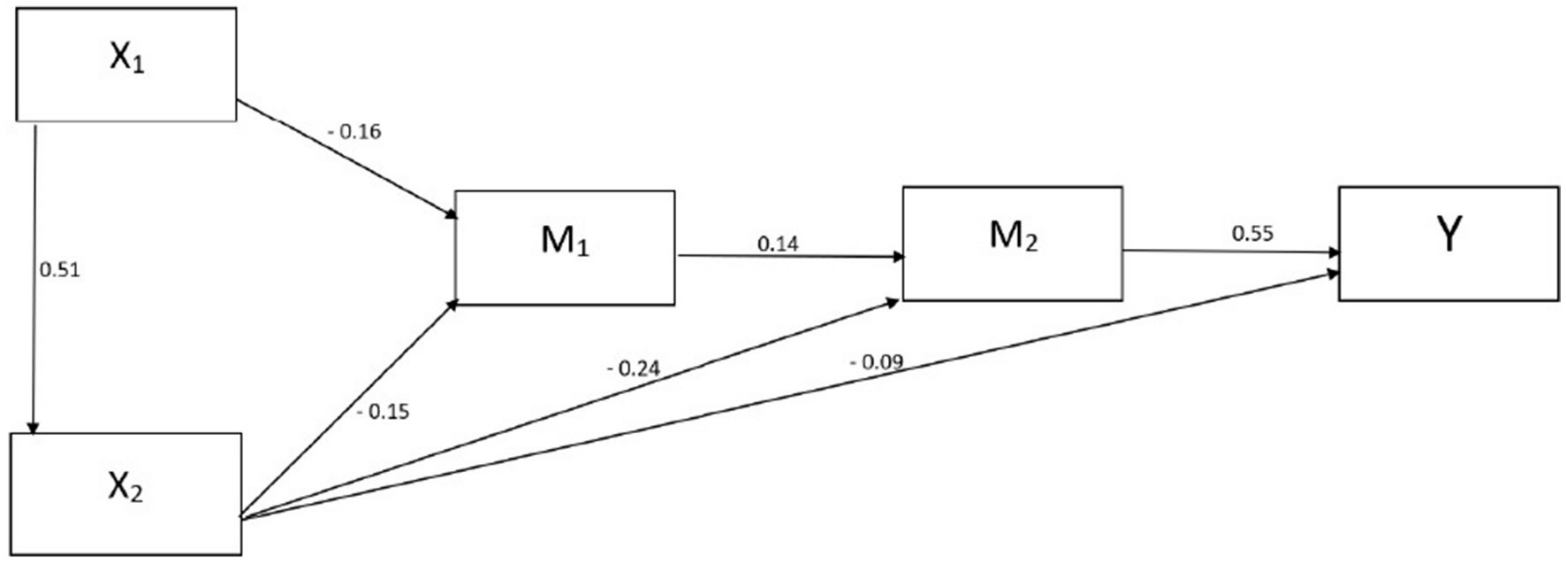
The summary of the hypothesis test's significant results [techno-insecurity (X_1_), techno-complexity (X_2_) desire to work from home (Y) the status sub-factor of Jahoda's latent deprivation model (M_1_), satisfaction with working from home (M_2_)].

The model we created is coherent with the studied data [$\chi_{\left(3\right)}^{2}$ =7.750 *p* = 0.05; CFI = 0.985, TLI = 0.950, RMSEA = 0.066, SRMR = 0.0240]; therefore, we considered it to be definitive and the basis for interpretation. Figure 2, Table 4 illustrate the results of the hypothesis tests.

**Table 4:** The examination of the mediation pathways underlying our hypotheses and the standardized effect between the variables tested in the model [techno-insecurity (X_1_), techno-complexity (X_2_) desire to work from home (Y), the status sub-factor of Jahoda's latent deprivation model (M_1_), satisfaction with working from home (M_2_)].

	**β-values**	**SE**	***p***	**CI (95%)**	
				**Lower**	**Upper**
**Direct effects**					
t-insecurity ← →t-complexity (X_1_ ← →X_2_)	0.509	0.048	0.001	0.404	0.598
t-insecurity →status (X_1_ →M_1_)	−0.161	0.062	0.01	−0.285	−0.04
t-complexity →status (X_2_ →M_1_)	−0.153	0.057	0.009	−0.258	−0.033
status →satisfaction with WFH (M_1_ →M_2_)	0.137	0.055	0.020	0.019	0.237
t-complexity →satisfaction with WFH (X_2_ →M_2_)	−0.243	0.054	0.001	−0.350	−0.138
t-complexity →desire to WFH (X_2_ →Y)	−0.095	0.048	0.047	−0.188	−0.001
Satisfaction with WFH →desire to WFH (M_2_ →Y)	0.547	0.041	0.002	0.456	0.619
**Indirect effects**					
H1: X2→M1 →M2 →Y (p2^*^p4^*^p5)	−0.468	0.272	0.012	−1.221	−0.073
H2: X2 →M2 →Y (p6^*^p5)	−5.445	1.393	0.001	−8.551	−2.972
H3: X1 →M1 →M2 →Y (p3^*^p4^*^p5)	−0.542	0.335	0.001	−1.472	−0.073
H4: X2 →M2 →Y (p8^*^p5)	−2.026	1.454	0.163	−4.966	0.913
Total indirect effect (TIE)	−7.524	1.655	0.001	−11.03	−4.502
Total effect (TE)	−11.40	2.209	0.001	−15.68	−6.977

Table 5 shows the summary results of the hypothesis analyses. Three out of the four hypotheses tested (H_1_, H_2_, H_3_) were confirmed, so it can be said that techno-complexity and techno-insecurity, although having a relatively small direct impact on the desire to work from home, presents harmful effects through numerous indirect ways. Our 4th hypothesis (H_4_) was rejected due to a lack of significance. The predictors presented in our model explain 33.7 per cent of the variance in the desire to work from home (Y) (R^2^ = 0.337 *p* = 0.02 SE = 0.042, CI 95% = 0.250–0.415).

**Table 5:** Summary results of the hypothesis analyses.

**Hypothesis**	**Result**
H1: We assume that techno-complexity (X2) reduces the desire to work from home (Y) through the status subfactor described in Jahoda's latent deprivation model (M1) and through satisfaction with WFH (M2). (p2^*^p4^*^p5)	Confirmed
H2: We assume that techno-insecurity (X1) reduces the desire to work from home (Y) through the status subfactor described in Jahoda's latent deprivation model (M1) and through satisfaction with WFH (M2). (p3^*^p4^*^p5)	Confirmed
H3: We assume that techno-complexity (X2) reduces the desire to work from home (Y) through satisfaction with WFH (M2). (p6^*^p5)	Confirmed
H4: We assume that techno-insecurity (X2) reduces the desire to work from home (Y) through satisfaction with WFH (M2). (p8^*^p5)	Rejected

## 4 Discussion

As a consequence of digitalization, the world of work is changing (Bresciani et al., 2021; Dabić et al., 2023). Working from home is becoming more popular among employees and organizations as well (Davis et al., 2020; Stefaniec et al., 2022). In our current study, we examined the indirect effects of technostress on the desire to work from home. Our research question is vital because there is a significant economic advantage for organizations to have employees working from home (Elst et al., 2017; Kuruzovich et al., 2021); however, the employees must not be forced to WFH, but they have a desire to do so. After all, the harmful organizational effects of authoritarian decisions have been demonstrated in research in the past decades (Lewin and Lippitt, 1938; Pellegrini and Scandura, 2008; Harms et al., 2018; Simon et al., 2021).

One of the significant adverse effects of digital work is the increasing emergence of technostress. In the past few years, since the spread of digital work, more and more studies have been written on this topic, so we can state that every subfactor of technostress negatively affects both the employees and the organizations (La Torre et al., 2020; Salazar-Concha et al., 2021). However, it has become essential to understand that technostress can have adverse effects not only directly but also indirectly.

To understand the effect of the various subfactors of technostress has on the desire to work from home, we also wanted to see how it affects the latent benefits of work. We assumed that technostress damages the experience of the status subfactor of Jahoda's latent deprivation model, which will worsen the desire to work from home.

We defined 4 hypotheses, in which we wanted to examine the indirect effect of techno-complexity and techno-insecurity on the desire to work from home through the status subfactor of Jahoda's latent deprivation model and the satisfaction with WFH. We approached our research questions by relying on a serial multiple mediation model.

We had to dismiss our 4th hypothesis (H_4_) based on our model (Table 4); thus, techno-insecurity does not directly harm the satisfaction with WFH and the desire to WFH. In contrast, our 3rd hypothesis (H_3_) was confirmed, which shows that the status subfactor mediates the negative relationship between techno-insecurity and satisfaction with WFH and the desire to work from home. This mediation path is logical given the knowledge of the variables since the techno-insecurity sub-factor measures the stress of the employee, who is afraid of losing their job or being demoted to a lower position because of digital technology or a more knowledgeable candidate (Ragu-Nathan et al., 2008). With the status subfactor of the latent deprivation model, we examine one's place in society (Paul and Batinic, 2010); therefore, we can state that the employee is afraid of losing their current status not just in the workplace but also in society due to the threat posed by digital technology, which will harm their satisfaction with WFH and negatively affect their desire to work from home.

On the one hand, the path through the status mediating variable (H_1_) has been confirmed, according to which the complexity of the new digital technology and the stress resulting from the difficulty of mastering it will endanger the employee's position, leading to a decrease in satisfaction and thus in desire. At the same time, techno-complexity directly harms the satisfaction of working from home (H_3_). Our model accounts for 33.7% of the variance of the desire to work from home, which can be considered a high value.

Previous studies have confirmed that different sub-factors of technostress affect job satisfaction in both offices (Tarafdar et al., 2010; Shi et al., 2023) and teleworking (Fernández-Fernández et al., 2023). Our own research, specifying these findings, confirmed that technostress has a detrimental effect on job satisfaction and WFH satisfaction. However, our research has also shown that it indirectly reduces the desire for WFH. In their previous research, Lansmann et al. (2023) identified several factors that predict this intention, such as segmentation preference (how important it is for a worker to set strict boundaries between work and private life), perceived productivity during mandated WFH or gender (female workers prefer this form of work). Other research published in the literature focuses exclusively on which variables positively predict the desire for WFH, such as higher income, educational attainment, supportive social norms, or control over WFH choice (Jain et al., 2022; Delbosc and Kent, 2023). Although previous studies have emphasized individual differences, by recognizing the importance of techno-complexity and techno-insecurity, we can get a more general picture of decreased WFH desire, which may make it easier for managers to help their employees and increase WFH intentions naturally.

### 4.1 Practical implications

With our model, we hoped to present that even though the subfactors of technostress have a minimal impact directly, they indirectly affect the desire to work from home; thus, it is an essential task for employers and managers to decrease its emergence. Of course, technostress can pose a serious problem not just while WFH but also in the office when the employees work with digital devices. At the same time, WFH is likely to be associated with higher technostress scores because it places parts of the work into a digital space that would not be necessary when working offline (such as communication between colleagues). Moreover, WFH can blur the boundaries of work time and space, further increasing the stress level experienced. Several effective organizational interventions can be established against technostress, such as literacy facilitation, technical support provision or technology involvement facilitation (Tarafdar et al., 2011). Other studies recommend practical advice for organizations to reduce technostress, such as reducing e-mail circulation, homogenizing digital platforms and increasing the frequency of IT training (Valta et al., 2021). Another effective way to cope with technostress is to reinforce employee gratitude through positive reframing (Garg et al., 2023).

It is vital to note, however, that the various organizational interventions affect the subfactors of technostress differently; thus, the leaders must react to the threat posed by techno-insecurity and techno-complexity in a specific manner. One of the most important ways to combat techno-insecurity is for managers to regularly reassure and calm their employees that their work is valuable to the organization and that modern technology will not take over their position. Supportive leadership, positive reinforcement, and recognition can have many other positive benefits in addition to reducing techno-insecurity, such as improving employee wellbeing and productivity (Suleman et al., 2021). Preventing techno-insecurity can be helped if the organization aims to foster a cooperative rather than a competitive culture. In these organizations, digitalization is seen as a shared goal, where the active participation of employees and the role modeling of managers help to achieve a long-term and sustainable culture (Turel and Gaudioso, 2018; Benlian, 2020).

Techno-complexity can be effectively prevented by allowing workers to learn the new technology introduced in workshops and training sessions so that they do not have to deal with the difficulties alone. Another effective solution could be to provide mentors for employees. Employees can request a mentor as a trusted contact person for guidance on digital and technical issues, who regularly provides tips and tricks for using ICTs, thereby lowering the inhibition threshold for asking questions (Chandra et al., 2019; Berger et al., 2023).

At the same time, our research shows that, in addition to preventing and reducing technostress, it is also necessary to strengthen the perception of status as a latent benefit of work in employees. In parallel with the reduction of techno-insecurity, the perception of status can be enhanced if managers emphasize to their employees the value of their work and their role in the organization. This may be particularly important when working from home, as the reduction in face-to-face encounters may reduce affective commitment (Simon et al., 2023) and question the employee's role in the organization.

### 4.2 Limitations

Certain limitations complicated our research. The research was conducted as a cross-sectional study using a self-administered online questionnaire. Thus, the respondents' subjective distortions could have impacted our results. The LAMB scale and the Technostress Creators scale had no validated Hungarian translation at this time. Our sample cannot be viewed as representative of the total Hungarian labor market, and there was no balance in the 14 analyzed sectors among the people who filled out our questionnaire.

### 4.3 Future work

In the future, we would like to expand our current research by examining other mediating variables through which the subfactors of technostress affect the desire to work from home. Furthermore, we would like to test our model not only with cross-sectional data but also with longitudinal data. With our research, we would like to focus on organizations effectively taking action against technostress, so we can expand our knowledge not just of its negative impact but also of how these impacts can be reduced. We hope that our findings on the indirect effects of technostress could contribute to developing long-term programs of assistance and intervention.

## 5 Conclusion

In our study, we wanted to explore how the various subfactors of technostress affect the desire to work from home because it is vital that organizations do not decree this method of work organization as a mandatory requirement but in line with employees' needs. For our research sample, we looked for office employees who, in the past 3 years, spent some of their work time WFH since digital work is more widespread among employees of high status and education. Our examination found that techno-insecurity and techno-complexity directly impact the desire to work from home only minimally. However, it significantly and vastly reduces the desire to work from home on several mediation paths of the latent deprivation model's status subfactor and through satisfaction with WFH. The results are intended to raise awareness among managers and practitioners of the importance of reducing technostress.

## Data availability statement

The original contributions presented in the study are publicly available. The datasets analyzed for this study can be found on the Open Science Framework: https://osf.io/yqtf2/?view_only=d5fdcce5ce9749ceb34fd8d66b43d8f7.

## Ethics statement

The study was approved by the Research Ethics Committee of the authors' institution (ELTE PPK; Reference number: 2022/94, Date: 25.02.2022). The studies were conducted in accordance with the local legislation and institutional requirements. The participants provided their written informed consent to participate in this study.

## Author contributions

AS: Conceptualization, Data curation, Formal analysis, Investigation, Methodology, Visualization, Writing – original draft, Writing – review & editing. BB: Conceptualization, Formal analysis, Methodology, Writing – review & editing. OR-F: Formal analysis, Methodology, Writing – review & editing. KF: Conceptualization, Supervision, Writing – review & editing. OP: Conceptualization, Supervision, Writing – review & editing. OK: Conceptualization, Supervision, Writing – review & editing.
